# Control Meets Inference: Using Network Control to Uncover the Behaviour of Opponents

**DOI:** 10.3390/e24050640

**Published:** 2022-05-02

**Authors:** Zhongqi Cai, Enrico Gerding, Markus Brede

**Affiliations:** School of Electronics and Computer Science, University of Southampton, Southampton SO17 1BJ, UK; eg@ecs.soton.ac.uk (E.G.); Markus.Brede@soton.ac.uk (M.B.)

**Keywords:** network inference, voting dynamics, complex networks, network control

## Abstract

Using observational data to infer the coupling structure or parameters in dynamical systems is important in many real-world applications. In this paper, we propose a framework of strategically influencing a dynamical process that generates observations with the aim of making hidden parameters more easily inferable. More specifically, we consider a model of networked agents who exchange opinions subject to voting dynamics. Agent dynamics are subject to peer influence and to the influence of two controllers. One of these controllers is treated as passive and we presume its influence is unknown. We then consider a scenario in which the other active controller attempts to infer the passive controller’s influence from observations. Moreover, we explore how the active controller can strategically deploy its own influence to manipulate the dynamics with the aim of accelerating the convergence of its estimates of the opponent. Along with benchmark cases we propose two heuristic algorithms for designing optimal influence allocations. We establish that the proposed algorithms accelerate the inference process by strategically interacting with the network dynamics. Investigating configurations in which optimal control is deployed. We first find that agents with higher degrees and larger opponent allocations are harder to predict. Second, even factoring in strategical allocations, opponent’s influence is typically the harder to predict the more degree-heterogeneous the social network.

## 1. Introduction

Revealing the network structure and thereafter reconstructing the ongoing network dynamics from observational data is the fundamentally inverse problem in network science [1,2]. As the reconstruction of complex networked systems from data observed in dynamical processes plays an essential role in practical applications aimed at the understanding and control of networked dynamics, it has attracted increasing attention in a wide range of research fields recently [3]. Prominent applications range from the discovering of genetic regulatory networks from gene expression data in computational biology  [4,5], uncovering functional or structural brain networks from sensed data in neuroscience [6,7], reconstructing contact networks from contagion data in epidemiology [8,9], to revealing hidden social connections from social media data on information cascades in social science [10,11]. In the typical setting investigated in the literature, observational data for reconstructing network structure and inferring parameters of dynamical processes are given as time series [3]. Most previous research has focused on the network reconstruction problem under the assumption that the entire time series of the network dynamics is accessible to ensure sufficient information is provided for accurate network inference. However, as investigated by the work of [8], in many real-world cases such as neuron cascades and epidemic spreading, the first stage of propagation is hard to measure and only a limited number of data points will be observed. Despite the experimental or technical limitations for data collection, obtaining high-precision estimations with less data is always desirable, especially when the measurement for the dynamical quantities is costly [12]. Motivated by the dilemma between the availability of observational data and the accuracy of inference, in this paper, we explore the issue of accelerating the convergence of inference by discovering more informative observational data. However, different from previous literature such as [8], we develop and explore a framework of how convergence of estimates can be accelerated through targeted interaction with the networked dynamics. Our framework thus supposes that the observer can influence the dynamical process on the network and we explore how such influence can be optimally deployed to improve the inference of unknown parameters of the dynamics.

To derive dynamical process parameters or reconstruct network topology from observational data, it is often necessary to draw on domain-specific expertise [3]. Here, we place the problem of speeding up inference in the context of opinion dynamics using the well-known competitive influence maximization framework [13,14], which studies the competition among external controllers who aim to maximally spread their opinions in the network through strategically distributing their influencing resources. Specifically, a common assumption while investigating the competitive influence maximization problem is that the external controllers are unaware of the strategy being used by their opponents during the competition. However, as, e.g., shown in [15], knowing the opponent’s strategy allows for a better design of influence allocations. For instance, in the setting of [15] when the controller has more resources than its opponent, a good strategy is to target the same agents as the opponent to shadow the opponent’s influence. Otherwise, the controller should avoid wasting resources and rather target agents not targeted by the opponent. Making use of such heuristics, however, presupposes knowledge of the opponent’s strategy. Moreover, as there are inherent time limits in many practical applications of competitive influence maximization [16,17], there may be limited time to learn from observation of opponents in many real-world settings. Indeed, the need of inferring the opponent’s behaviour in a short time frame is observed in many real-world contexts, such as finding out the source of fake news as soon as possible in the social network to stop it from spreading [18], analysing the provenance of extreme opinions to prevent radicalization [19], and uncovering the strategy of the opposing political parties before a given deadline to gain advantages in the election [20]. Therefore, accelerating the inference to obtain better estimates of opponent’s strategies from dynamical data within a short time frame is an important problem relevant to competitive influence maximization.

To be more concrete, in this paper we explore the problem of opponent strategy inference in the setting of the competitive voting dynamics as studied in [15,16,21]. This choice is motivated by the popularity of the voter model in opinion dynamics as well as its high levels of tractability [22]. Specifically, in the voting dynamics, opinions are represented as binary variables, and each agent in the network holds one of two opinions. On top of the internal agents, following the work of [15,16,23], the external controllers exert their influence on the network by building unidirectional connections with agents, in which the intensity of their targeting are represented by link weights. The opinion propagates according to the rules that agents flip their opinion states with probabilities proportional to the number of agents with opposing opinions and link weights from opposing controllers [21]. The problem we are interested in is that one of the controllers can change its control allocations to accelerate its learning of the opposing controllers targeting through observation of the voting dynamics.

Since we model the way of exerting influence from external controllers by building unidirectional connections with agents in the network, the connections from the external controller can also be viewed as edges that constitute part of the network topology. Therefore, our research problem of opponent strategy inference is closely related to the topic of network structure inference. There is rich literature in the field of reconstructing network structure from information flows [3], and a detailed review of the related work within the domains of epidemiology and information spreading is given in Section 2. Most relevant to our modelling approach, [11,24,25] infer the network topology from time series of binary-state data. More specifically, [11,24] treat the connections between agents as binary variables, and transform the network inference problem to identifying the existence of binary links between agents. Hence, these approaches are unsuitable to infer continuous interaction intensity between agents and from the external controllers. Further to the works of [11,24], Chen and Lai [25] remove the binary restriction and consider the network inference problem in a continuous space by developing a data-driven framework to predict link weights. Nevertheless, none of these works investigate the network inference problem from the perspective of manipulating the opinion diffusion process to accelerate the convergence of estimation, which is an important lever if one wants to obtain an estimate with an accuracy guarantee within a short and limited observation time.

To address the current gaps in accelerating the convergence of inference, in this paper, we follow the setting of our previous work [26], in which we relate the problem of accelerating opponent strategy inference with network control. By doing so, we assume an active strategic controller who tries to minimise the uncertainty of inference of an opponent’s strategy by optimally allocating its control resources to agents in the network based on the voter model. In other words, we explore how a controller can modify network dynamics such that the influence of opponents becomes easier to identify. Note that we always assume only limited resources are available for the active controller to interfere with the network dynamics, since for most real-world applications [14,27], there are natural resource constraints.

In the following, our main interest is in designing heuristic algorithms for allocating limited resources of the active controller. This will enable the generation of more informative observational data during the opinion propagation process and thereby accelerate the convergence of the estimations of the opponent’s strategy. Our paper is based on results that have previously been presented at the Conference on Complex Networks and their Applications 2021 [26]. Beyond a more detailed exposition of the problem, we additionally extend the previously presented results in two important ways. First, we discuss the ability to predict for an optimizing controller in the face of different opponent strategies. Second, we propose an improved algorithm (which we name the two-step-ahead optimization). In contrast to what we presented in [26] this new method also accounts for indirect influence between agents in the optimization of resource allocations.

Our main contributions are as follows: First, before our work of [26], the network inference in the field of information spreads has never been studied from the perspective of strategically interacting with the opinion dynamics to speed up the process of inference. In this paper, we extend the results from [26] and provide a systematic investigation of how to optimally deploy resources in order to maximally accelerate the opponent strategy inference. Second, we model the opinion propagation process for an individual agent in the network as a non-homogeneous Markov chain and further derive estimators of the opponent’s strategy via maximum likelihood estimation. We also provide uncertainty quantification of our estimators by using the variance deduced from the expectation of the second-order derivative of the likelihood function. This, in turn, is used to inform decisions on the optimal allocations and understand the process of inference acceleration. Third, we develop several heuristic algorithms for speeding up opponent strategy inference via minimizing the variance of estimators, and test the effectiveness of our algorithms in numerical experiments.

The key findings of our work are as follows. First, we demonstrate that it is possible to accelerate the inference process by strategically interacting with the network dynamics. Second, we consider two settings: One is accelerating the inference of the opponent strategy at a single node, when only the inferred node is controllable. The other is minimizing the variance of the opponent influence at the inferred node when both the inferred node and also its neighbours are controllable. In the first setting, we find that the optimized resource allocation is inversely proportional to the sum of neighbouring opinion states. In the second setting, we observe two regimes of the optimized resource allocations based on varying amounts of available resources for the active controller. If the active controller has very limited resources, then it should target the inferred node only. In contrast, if resources are large, a better strategy is to not target the inferred node, but instead focus only on neighbouring nodes. Third, in the scenario of inferring opponent strategies over entire networks, strategic allocations become increasingly important as more resources are available for the active controller. We also find that nodes with lower degrees and targeted with smaller amount of resources by the opponent will generally have a smaller variance in inference.

The structure of this paper is as follows. Section 2 gives an overview over the state-of-the-art on network inference in the field of reconstructing network structure. Section 3 formalises the problem of accelerating opponent strategy inference for the voter model and presents heuristics for solving the opponent strategy inference problem. Section 4 shows the corresponding results after applying the heuristics. Section 5 summarises the main findings and discusses some ideas for future work.

## 2. Related Work

As our study is based on the opinion dynamics, we first provide an overview of existing research from the closely related domain of reconstructing network structure from epidemiology and information spreads. Starting from the seminal work of Gomez-Rodriguez et al. [28], inferring networks using maximum likelihood methods in this area has been extensively explored in a variety of scenarios. In Gomez-Rodriguez et al. [28], the authors treat network structure inference as a binary optimization problem (i.e., whether or not there is an edge between two agents) and propose the NetInf algorithm based on the maximization of the likelihood of the observed cascades in a progressive cascade model [29], where the opinion propagation occurs as a one-off process. To improve the performance of the NetInf algorithm in the progressive cascade model, Rodriguez and Schölkopf [30] propose the MultiTree algorithm by including all directed trees in the optimization. In addition, algorithms have been developed to infer the intensity of connections by Braunstein et al. [8] based on the susceptible–infected–recovered model, which is also a progressive cascade model. Moreover, some other works have incorporated prior knowledge about the network structure (e.g., sparsity [31], motif frequency [32], or community structure [33]) to improve the performance of network inference given limited amounts of data.

In order to incorporate uncertainty in inference, several other works employ Bayesian inference using Markov chain Monte Carlo methods. Early works in the domain of epidemiology [34,35] treat the network model (e.g., an Erdős-Rényi random graph, or a scale-free network [36]) as known, and use Bayesian inference to discover the network model parameters as well as diffusion parameters (e.g., the infection rate). However, the assumption of knowing the network model is too restrictive and, in most cases, inference of structural information is necessary. The most representative work of using Bayesian inference to reconstruct network structure from information cascades is the work by Gray et al. [2], which has improved estimates of network structure, especially in the presence of noise or missing data, and is also based on the progressive cascade model. However, their work assumes that the adjacency matrix of the underlying graph is binary, and it is therefore not suitable for inferring the intensity of connections.

Most of the above-mentioned works reconstruct network structure from observations of information cascades or infection trees and are based on progressive cascade models. However, the assumption of the progressive cascade models that once an agent gets infected, its state will remain unchanged is inappropriate for modelling opinion dynamics, as opinion states can be switched back and forth in most cases. The exceptions that explore network structure based on non-progressive models (e.g., the voter model, the suspicious-infected-suspicious (SIS) model, the Ising model) are Barbillon et al. [9], Li et al. [24], Chen and Lai [25] and Zhang et al. [11]. In more detail, Barbillon et al. [9] apply the matrix-tree theorem to infer the network structure based on a susceptible–infected–susceptible model. To maintain the information cascades as a directed acyclic graph as works based on progressive cascade models, the information propagation has been encoded as a matrix with m×n dimensions where *n* represents the number of individuals and *m* is the length of time series. Unlike Barbillon et al. [9] and all works based on progressive cascade models which need input sequences of agents with infection times sorted from a root and monotonically increasing, the works by Li et al. [24], Chen and Lai [25] and Zhang et al. [11] reconstruct network structure from observations of binary-state dynamics. In more detail, Li et al. [24] translate the network structure inference into a sparse signal reconstruction problem by linearization and solve it via convex optimization. Moreover, Chen and Lai [25] develop a model combining compressive sensing and a clustering algorithm for network reconstruction. However, the above works only consider unidirectional infection (e.g., in the SIS model, if a susceptible node is in contact with an infected node, it will be infected according to a certain probability. Nevertheless, an infected node will not change to the susceptible state due to the contact with another susceptible node but according to a systematic recovery rate). Instead, Zhang et al. [11] solve the network inference problem by expectation maximization with a focus on the setting that two states are equivalent (as, e.g., in the voter model) and utilize bidirectional dynamics to calculate transition probabilities to reduce the amount of data needed for accurate estimation. However, this work treats an edge as a binary variable (i.e., the existence or absence of a link between two nodes), and it is not suitable for inferring the link weight between two agents.

To summarise, most works in the field of epidemiology and information propagation infer network structure from information cascades or infection trees which are identical to directed acyclic graphs, and are not applicable to situations where opinions can be changed back and forth. Moreover, none of these works combines network control with the network structure inference where external controllers can interact with the intrinsic dynamics of opinion propagation to elicit more information during inference.

## 3. Model Description and Methods

We consider a population of *N* agents exchanging opinions through a social network *G*. The social connections between agents are represented by an adjacency matrix ${W=\{w_{ij}\}}_{i,j=1}^{N}$, with $w_{ij}=1$ indicating the existence of a social link between agent *i* and agent *j* and $w_{ij}=0$ otherwise. Note that agent *i* and *j* are called neighbours if there is a link between them. Moreover, we assume that each of the *N* agents holds a binary opinion at time *t* denoted as $s_{i}\left(t\right)\in \left\{0,1\right\}$ (i=1,…,N). In addition, opinion propagation through the social network follows the classic voter model [22] where agents copy one of their neighbours’ opinions according to a probability proportional to the weight of social connections.

On top of the classic voter model, following the works of [15,16,21], we consider the existence of two external controllers, named controller *A* and *B*. In more detail, controller *A* and *B* are zealots who have fixed opinions $s_{A}\left(t\right)=1$ and $s_{B}\left(t\right)=0$ for ∀t≥0. By building unidirectional and non-negatively weighted links $a_{i}\left(t\right)\geq 0$ and $b_{i}\left(t\right)\geq 0$ to agent *i* at time *t*, the two external controllers *A* and *B* exert their influence on the social network and therefore interact with the intrinsic opinion dynamics. Here, the sum of the link weights are subject to budget constraints, i.e., $\sum_{N}a_{i}\left(t\right)\leq b_{A}$ and $\sum_{N}b_{i}\left(t\right)\leq b_{B}$, where $b_{A}$ and $b_{B}$ are the total resources available to controller *A* and *B* respectively. The weighted links $a_{i}\left(t\right)$ and $b_{i}\left(t\right)$ are also taken into consideration in the opinion updating process. In more detail, we assume a parallel and discrete-time opinion updating for the whole population as follows: at time *t*, agent *i* (i=1,…,N) updates its opinion to $s_{i}\left(t+1\right)=1$ with probability

(1) $$Pr\left(s_{i}\left(t+1\right)=1\right)=\frac{a_{i}\left(t\right)+\sum_{j}s_{j}\left(t\right)w_{ji}}{\sum_{\ell =1}^{N}w_{\ell i}+a_{i}\left(t\right)+b_{i}\left(t\right)},$$

and to $s_{i}\left(t+1\right)=0$ with probability

(2) $$Pr\left(s_{i}\left(t+1\right)=0\right)=\frac{b_{i}\left(t\right)+\sum_{j}\left(1-s_{j}\left(t\right)\right)w_{ji}}{\sum_{\ell =1}^{N}w_{\ell i}+a_{i}\left(t\right)+b_{i}\left(t\right)}.$$

**Figure 1:**
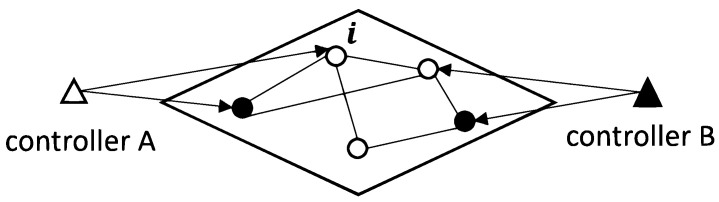
Schematic diagram of how controllers interact with the opinion dynamics and how agents update their opinions. Triangles stand for controllers and agents are represented by circles. Black and blank symbols indicate that the agents (or controllers) are holding opinion 1 or 0, respectively. The lines between agents correspond to the social connections. External controllers A and B influence opinion dynamics by building unidirectional links to agents in the networks. Assuming unity link weights from the neighbours and controllers, in the next time step, agent *i* will have probability 3/4 to stay in opinion 1 and probability 1/4 to flip its opinion.

From the equations of $Pr(s_{i}\left(t+1\right)=1)$ and $Pr(s_{i}\left(t+1\right)=0)$, note that the opinion transition probabilities are determined only by the neighbouring states of the updated agent and the weighted links from the controllers, and they are independent of the current opinion of the updated agent. For a better understanding of our framework an illustration is given in Figure 1. Take agent *i* as an example and assume unit-strength connections between agents and from the controllers. Agent *i* in Figure 1 is linked with three other agents (one of which holds opinion 0 and two who hold opinion 1), and is targeted by controller *A*. Therefore, in the next update, agent *i* will have probability 3/4 to stay in opinion 1 and probability 1/4 to flip its opinion to 0.

From the perspective of external controllers, they aim to maximize their influence by strategically allocating resources to agents in the network under the context of competitive influence maximization. According to [15], knowing the opponent’s strategies allows for an efficient budget allocation to maximise influence. However, even though it may be possible to directly observe agents’ opinions at each time step, observing the strategies of controllers, i.e., if an agent is targeted by the external controller, or even how strong the intensity of influence from the controllers is, are often very challenging [37]. For instance, considering opinion propagation on social media, as the users adopt a new opinion, they may post it without mentioning the source. Thus, we only observe the time when the user’s opinion is changed, but not who it was influenced by.

To solve this problem of opponent-strategy reconstruction from observable data, we model the updating process of agent *i* (i=1,…,N) as a non-homogeneous Markov chain [38] where the Markov property is retained but the transition probabilities $Pr(s_{i}\left(t+1\right)=1)$ and $Pr(s_{i}\left(t+1\right)=0)$ depend on time. Further to this formalization, we assume an active controller *A* infers the strategy of the passive and constant controller *B* who has fixed budget allocations (i.e., $b_{i}\left(t\right)=b_{i}\left(0\right)$, i=1,…,N, ∀t≥0) from the time series of agents’ opinion changes. Here, the time series are given by a matrix $S={\left[s_{i}\left(t\right)\right]}_{N\times T}$ where *T* is the length of the observation period. In other words, while updating the voting dynamics, we obtain a data matrix *S* with *N* rows and *T* columns in which each row of *S* denotes the binary opinion dynamics of an agents over an observation period of length *T*. Taking the data matrix *S* as an input, we are interested in decoding the unknown parameters $b_{i}\left(t\right)$ (referred to as $b_{i}$ in the following) from the input. Given the transition probabilities $Pr(s_{i}\left(t+1\right)=1)$ and $Pr(s_{i}\left(t+1\right)=0)$ of the opinion flow between agents in the existence of controller, a commonly-used method for solving such parametric inference is maximum-likelihood estimation (MLE) [28]. Specifically, replacing $s_{i}\left(t+1\right)$ and $s_{j}\left(t\right)$ with data actually observed along time series from 0 to *T* yields the log-likelihood function of agent *i*

(3) $$\begin{array}{cc} L_{i}\left(T\right)=\sum_{t\in [0,T-1]} & [s_{i}\left(t+1\right)\log\frac{a_{i}\left(t\right)+\sum_{j}w_{ji}s_{j}\left(t\right)}{a_{i}\left(t\right)+b_{i}+k_{i}}+\\ \; & \left(1-s_{i}\left(t+1\right)\right)\log(\frac{b_{i}+\sum_{j}w_{ji}\left(1-s_{j}\left(t\right)\right)}{a_{i}\left(t\right)+b_{i}+k_{i}})] \end{array}$$

where $k_{i}$ is the degree of node *i*, i.e., $k_{i}=\sum_{\ell =1}^{N}w_{\ell i}$. This log-likelihood function gives the likelihood of observing an agent’s time series, given the parameter $b_{i}$. Depending on the opinion states in the next step $s_{i}\left(t+1\right)$, either $Pr(s_{i}\left(t+1\right)=1)$ or $Pr(s_{i}\left(t+1\right)=0$ is taken into account in the log-likelihood function of Equation  (3). We then estimate the budget allocations of controller *B* to be the values $b_{i}$ that are most likely to generate the given data matrix *S* after *T* observations. Therefore, we maximize the log-likelihood function $L_{i}\left(T\right)$ in Equation (3) with respect to the budget allocations of controller *B* to obtain an estimate of $b_{i}$, denoted as ${\hat{b}}_{i}$ in the following.

According to the consistency of maximum likelihood estimates [39], for a sufficiently large dataset, the estimator asymptotically converges to the true value. However, in this paper, we are interested in the problem of whether the observations of opinion states can be improved by interfering with the opinion dynamics so that we will obtain good-fit estimates within limited observations. To achieve this, instead of passively observing, we assume the controller *A* is an active controller who strategically allocates its resources to accelerate the inference of the strategy of its opponent (i.e., $b_{i}$, 1≤i≤N). To evaluate the goodness of fit of the inference obtained from MLE, a commonly-used measurement is the Fisher information [40]. Specifically, Fisher information is used to test if the maximum likelihood estimators are aligned with the dataset and to derive a measure of dispersion between the true value and the estimator. Following [40], the Fisher information $I(b_{i},T)$ about $b_{i}$ is given by the expectation of second-order partial derivative of Equation (3) with respect to $b_{i}$, which is given by

(4) $$\begin{array}{c} \begin{array}{cc} I(b_{i},T) & =E\left[\frac{\partial^{2}}{\partial b_{i}^{2}}L_{i}\left(T\right)\right]=-\sum_{t\in [0,T-1]}\frac{a_{i}\left(t\right)+\sum_{j}w_{ji}s_{j}\left(t\right)}{{\left(a_{i}\left(t\right)+b_{i}+k_{i}\right)}^{2}\left(k_{i}+b_{i}-\sum_{j}w_{ji}s_{j}\left(t\right)\right)}\\ \; & =\sum_{t\in [0,T-1]}\left[{\left(a_{i}\left(t\right)+b_{i}+k_{i}\right)}^{-2}-{\left(\left(a_{i}\left(t\right)+b_{i}+k_{i}\right)\left(k_{i}+b_{i}-\sum_{j}w_{ji}s_{j}\left(t\right)\right)\right)}^{-1}\right]. \end{array} \end{array}$$

For ease of exposition, let

$$\beta_{i}\left(t\right)={\left[\left(a_{i}\left(t\right)+k_{i}+b_{i}\right)\left(k_{i}+b_{i}-\sum_{j}w_{ji}s_{j}\left(t\right)\right)\right]}^{-1},$$

and

$$\Psi_{i}\left(t\right)={\left(a_{i}\left(t\right)+k_{i}+b_{i}\right)}^{-2}.$$

Given this, Equation (4) can be written as

$$I\left(b_{i},T\right)=\sum_{t\in [0,T-1]}\left(\Psi_{i}\left(t\right)-\beta_{i}\left(t\right)\right).$$

Moreover, in Equation (4) we have,

$$\frac{a_{i}\left(t\right)+\sum_{j}w_{ji}s_{j}\left(t\right)}{{\left(a_{i}\left(t\right)+b_{i}+k_{i}\right)}^{2}\left(k_{i}+b_{i}-\sum_{j}w_{ji}s_{j}\left(t\right)\right)}\geq 0.$$

Correspondingly, the negative sum of the above equation over *t* from 0 to T−1 is non-positive, and will decrease as the length of observation *T* increases. Hence, the Fisher information $I(b_{i},T)$ is also non-positive and monotonously decreasing as *T* increases.

As mentioned above, knowledge of the Fisher information is used to determine whether the maximum likelihood estimator is close to the true value. Specifically, for a large enough sample (i.e., T→∞), the maximum likelihood estimator ${\hat{b}}_{i}$ converges in distribution of a normal distribution to the true value $b_{i}$ [39], i.e.,

(5) $$\left({\hat{b}}_{i}-b_{i}\right)\overset{D}{\to }N\left(0,-{I(b_{i},T)}^{-1}\right),\;\mathrm{as}\;T\to \infty$$

where $N(0,-{I(b_{i},T)}^{-1})$ stands for a normal distribution with mean μ=0 and variance $\sigma^{2}\left(b_{i},T\right)=-{I(b_{i},T)}^{-1}$ for agent *i*. As the Fisher information is non-positive and monotonously decreasing along observations, the variance is always positive and, after a long period of observations, we will obtain more information and produce an estimator ${\hat{b}}_{i}$ closer to the true value $b_{i}$. Moreover, by taking the first order partial derivative of $\sigma^{2}\left(b_{i},T\right)$ with respect to $b_{i}$, one obtains

(6) $$\begin{array}{c} \begin{array}{cc} \frac{\partial \sigma^{2}\left(b_{i},T\right)}{\partial b_{i}} & =\frac{\partial \{-{I(b_{i},T)}^{-1}\}}{\partial I(b_{i},T)}\frac{\partial I(b_{i},T)}{\partial b_{i}}\\ \; & ={I(b_{i},T)}^{-2}\sum_{t\in [0,T-1]}\left[\frac{\left(a_{i}\left(t\right)+\sum_{j}w_{ji}s_{j}\left(t\right)\right)\left(a_{i}\left(t\right)+3\left(b_{i}+k_{i}\right)-2\sum_{j}w_{ji}s_{j}\left(t\right)\right)}{{\left(a_{i}\left(t\right)+b_{i}+k_{i}\right)}^{3}{\left(b_{i}+k-\sum_{j}w_{ji}s_{j}\left(t\right)\right)}^{2}}\right]\geq 0, \end{array} \end{array}$$

and we find that the variance is monotonously increasing with the increase of $b_{i}$ regardless the values of $a_{i}$ and $s_{i}$. Note that the variance in Equation (5) is calculated from Fisher information at the true value. As the true value of $b_{i}$ is unknown, in practical calculations we later replace the true value of $b_{i}$ with ${\hat{b}}_{i}$ to calculate the estimated variance ${\hat{\sigma }}^{2}\left({\hat{b}}_{i},T\right)$.

By introducing the Fisher information, we transform the problem of accelerating opponent strategy inference by interacting with the opinion dynamics into strategically deploying the budget of controller *A* to maximally decrease the variance of estimates. As the Fisher information can be represented in a recursive way, where the Fisher information at time *T* is calculated by Fisher information at time T−1 plus two additional terms, the variance can also be calculated recursively via

(7) $${\hat{\sigma }}^{2}\left({\hat{b}}_{i},T\right)=-{I({\hat{b}}_{i},T)}^{-1}=-{\left[I\left({\hat{b}}_{i},T-1\right)+{\hat{\Psi }}_{i}\left(T-1\right)-{\hat{\beta }}_{i}\left(T-1\right)\right]}^{-1},$$

where ${\hat{\beta }}_{i}\left(t\right)={\left[\left(k_{i}+{\hat{b}}_{i}-\sum_{j}w_{ji}s_{j}\left(t\right)\right)\left(a_{i}\left(t\right)+k_{i}+{\hat{b}}_{i}\right)\right]}^{-1}$, ${\hat{\Psi }}_{i}\left(t\right)={\left(a_{i}\left(t\right)+k_{i}+{\hat{b}}_{i}\right)}^{-2}$ and ${\hat{\sigma }}^{2}\left({\hat{b}}_{i},t+1\right)$ represents the expected variance at time t+1.

Inspired by the recursive expression for the variance in Equation (7), we propose two types of heuristics in which we explore configurations of the budget allocations of controller *A* at time *t* for node *i* (i.e., $a_{i}\left(t\right)$, i=1,…,N) to maximally decrease the expected variance of the estimators in future updates. Because of the combinatorics involved when dealing with arbitrary numbers of updates, we limit considerations to looking one or two steps ahead and correspondingly label the resulting heuristics *one-step-ahead optimization* and *two-step-ahead optimization*. Our strategy here is as follows. At time *t*, controller *A* has an estimate of the influence of controller *B* and an estimate of the variance around it. It then allocates its influence in such a way as to minimize the expected variance of its next estimate either one or two updating steps in the future.

In the following, we first give the formalized expressions of minimizing the variance of a single estimator ${\hat{b}}_{i}$ via optimizing the budget allocation on a single node *i* in the one-step-ahead and two-step-ahead scenarios, respectively. The extensions of these two heuristics will be further discussed in Section 4 in which we consider to optimize the budget allocations over multiple nodes to minimize the sum of variance for the entire network.

### 3.1. One-Step-Ahead Optimization

Specifically, for the one-step-ahead optimization scenario, the argument of the objective function through which we aim to minimize the one-step-ahead variance of estimator ${\hat{b}}_{i}$ is

(8) $$\begin{array}{cc} a_{i}^{*}\left(t\right) & =\arg\min{\hat{\sigma }}^{2}\left({\hat{b}}_{i},t+1\right)=\arg\min-{I({\hat{b}}_{i},t+1)}^{-1}\\ \; & =\arg\min-{\left[I\left({\hat{b}}_{i},t\right)+{\hat{\Psi }}_{i}\left(t\right)-{\hat{\beta }}_{i}\left(t\right)\right]}^{-1} \end{array}$$

where $a_{i}^{*}\left(t\right)$ is the optimized budget allocation for controller *A* at time *t* in order to minimize the expected variance at time t+1. Analogous to Equation  (7), we have ${\hat{\beta }}_{i}\left(t\right)={\left[\left(k_{i}+{\hat{b}}_{i}-\sum_{j}w_{ji}s_{j}\left(t\right)\right)\left(a_{i}\left(t\right)+k_{i}+{\hat{b}}_{i}\right)\right]}^{-1}$ and ${\hat{\Psi }}_{i}\left(t\right)={\left(a_{i}\left(t\right)+k_{i}+{\hat{b}}_{i}\right)}^{-2}$.

To define an experimental setup, we focus on obtaining a step-wise optimized budget allocation $a_{i}^{*}\left(t\right)$ for node *i* which can differ at each time step *t*, while fixing other nodes’ budget allocations as $a_{f}$. The one-step-ahead optimization algorithm then proceeds according to the following steps:

(i) To satisfy the premise of enough samples before using the Fisher information to calculate the variance of a maximum likelihood estimator, we let controller *A* target all nodes equally with fixed budget allocation $a_{f}$ for the first *m* updates and record the likelihood at time *m* as $L_{i}\left(m\right)$.
(ii) If the current updating step *t* is less than the length of total time series *T*, we calculate the current estimator ${\hat{b}}_{i}$ by maximizing the likelihood function $L_{i}\left(t\right)$ with respect to $b_{i}$ and evaluate the Fisher information $I({\hat{b}}_{i},t)$. Then, we calculate the expectation of the variance defined in Equation (8). Next, we obtain the optimized $a_{i}^{*}\left(t\right)$ by applying the interior point optimization algorithm [41]. Finally, we update the network with a new assignment of $a_{i}^{*}\left(t\right)$ and simulate the stochastic voting dynamics to gain the next-step states for all nodes.
(iii) The procedure is terminated when a fixed number of observations *T* have been made.

This procedure is more formally presented in Algorithm  1. The main body of Algorithm 1 (lines 3–7) corresponds to step (ii). After applying Algorithm 1, we obtain a sequence of $a_{i}^{*}\left(t\right)$ where m≤t≤T. Note that the initial states of agents are generated randomly to ensure that 50% of the initial opinions of agents are 0 or 1.
**Algorithm 1: **One-step-ahead optimization 
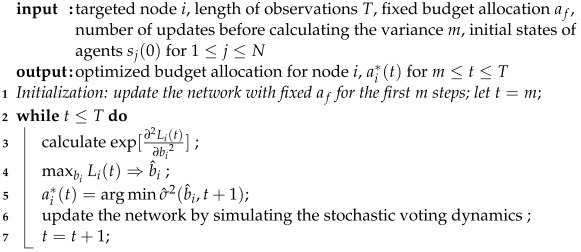



### 3.2. Two-Step-Ahead Optimization

For the two-step-ahead optimization scenario, we label the optimized budget allocations for node *i* at time *t* and t+1 as $a_{i}^{*}\left(t\right)$ and $a_{i}^{*}\left(t+1\right)$. Then, the objective function for minimizing the two-step-ahead variance is calculated by the expected negatively inverse Fisher information two steps ahead given by:

(9) $$\begin{array}{c} \begin{array}{cc} \; & \left\{a_{i}^{*}\left(t\right),a_{i}^{*}\left(t+1\right)\right\}=\arg\min{\hat{\sigma }}^{2}\left({\hat{b}}_{i},t+2\right)\approx \arg\min-{I({\hat{b}}_{i},t+2)}^{-1}\\ \; & =\arg\min-{\left[I\left({\hat{b}}_{i},t\right)+E\left[s_{i}\left(t+1\right)\left({\hat{\Psi }}_{i}\left(t\right)+{\hat{\Psi }}_{i}\left(t+1\right)\right)+{\bar{s}}_{i}\left(t+1\right)\left({\hat{\Upsilon }}_{i}\left(t\right)+{\hat{\Upsilon }}_{i}\left(t+1\right)\right)\right]\right]}^{-1}\\ \; & =\arg\min-[I\left({\hat{b}}_{i},t\right)+Pr\left(s_{i}\left(t+1\right)=1\right)Pr\left(s_{i}\left(t+2\right)=1\right)\left({\hat{\Psi }}_{i}\left(t\right)+{\hat{\Psi }}_{i}\left(t+1\right)\right)\\ \; & \;\;\;+Pr\left(s_{i}\left(t+1\right)=0\right)Pr\left(s_{i}\left(t+2\right)=0\right)\left({\hat{\Upsilon }}_{i}\left(t\right)+{\hat{\Upsilon }}_{i}\left(t+1\right)\right)\\ \; & \;\;\;+Pr\left(s_{i}\left(t+1\right)=1\right)Pr\left(s_{i}\left(t+2\right)=0\right)\left({\hat{\Psi }}_{i}\left(t\right)+{\hat{\Upsilon }}_{i}\left(t+1\right)\right)\\ \; & \;\;\;+Pr\left(s_{i}\left(t+1\right)=0\right)Pr\left(s_{i}\left(t+2\right)=1\right)\left({\hat{\Upsilon }}_{i}\left(t\right)+{\hat{\Psi }}_{i}\left(t+1\right)\right){]}^{-1} \end{array} \end{array}$$

where

$${\bar{s}}_{i}\left(t+1\right)=1-s_{i}\left(t+1\right),$$

$${\hat{\Upsilon }}_{i}\left(t\right)=-\frac{\left(a_{i}\left(t\right)+\sum_{j}w_{ji}s_{j}\left(t\right)\right)\left(a_{i}\left(t\right)+2k_{i}-\sum_{j}w_{ji}s_{j}\left(t\right)+2{\hat{b}}_{i}\right)}{{\left(a_{i}\left(t\right)+k_{i}+{\hat{b}}_{i}\right)}^{2}{\left(k_{i}-\sum_{j}w_{ji}s_{j}\left(t\right)+{\hat{b}}_{i}\right)}^{2}},$$

$${\hat{\Psi }}_{i}\left(t\right)={\left(a_{i}\left(t\right)+k_{i}+{\hat{b}}_{i}\right)}^{-2}.$$

Note that the probabilities of agent *i* having opinion 1 or 0 at the current time step are dependent on its neighbouring states at the previous time step. As in the one-step-ahead procedure, when performing the optimization of Equation (9), $s_{i}\left(t\right)$ for 1≤i≤N are known. Therefore, the expressions for $Pr(s_{i}\left(t+1\right)=1)$ and $Pr(s_{i}\left(t+1\right)=0)$ only contain one unknown parameter, which is $a_{i}\left(t\right)$. However, in the expressions of $Pr(s_{i}\left(t+2\right)=1)$ and $Pr(s_{i}\left(t+2\right)=0)$, the sum of their respective neighbouring opinions $\sum_{j}w_{ji}s_{j}\left(t+1\right)$ are unknown, and thus the full expressions for $Pr(s_{i}\left(t+2\right)=1)$ and $Pr(s_{i}\left(t+2\right)=0)$ are obtained via applying the law of total probability

(10) $$\begin{array}{c} \begin{array}{cc} Pr(s_{i}\left(t+2\right)=1) & =\sum_{m=0,\ldots ,k_{i}}Pr\left(s_{i}\left(t+2\right)=1|\sum_{j}w_{ji}s_{j}\left(t+1\right)=m\right)Pr\left(\sum_{j}w_{ji}s_{j}\left(t+1\right)=m\right)\\ & =\sum_{m=0,\ldots ,k_{i}}\frac{a_{i}\left(t+1\right)+m}{a_{i}\left(t+1\right)+b_{i}+k_{i}}Pr\left(\sum_{j}w_{ji}s_{j}\left(t+1\right)=m\right)\\ Pr(s_{i}\left(t+2\right)=0) & =1-Pr(s_{i}\left(t+2\right)=1), \end{array} \end{array}$$

where

(11) $$Pr\left(\sum_{j}w_{ij}s_{j}\left(t+1\right)=m\right)=\sum_{\rho =1}^{l}\prod_{j\in c_{\rho }}Pr\left(s_{j}\left(t+1\right)=1\right)\prod_{j\in (Nei\left(i\right)\backslash c_{\rho })}Pr\left(s_{j}\left(t+1\right)=0\right).$$

In the above, *l* stands for the number of combinations leading to $\sum_{j}w_{ij}s_{j}\left(t+1\right)=m$ and the elements of $C=\{c_{1},\ldots ,c_{l}\}$, represented as $c_{\rho }$ for 1≤ρ≤l, indicate all possible combinations of the neighbourhood of node *i* adding up to *m* at time t+1. If we denote the neighbourhood of node *i* as Nei(i), then $Nei\left(i\right)\backslash c_{\rho }$ returns the set of elements in Nei(i) but not in $c_{\rho }$. Inserting Equations (10) and (11) into Equation (9) yields the full expression for the goal function. The optimization procedure for the two-step-ahead scenario follows along the lines of Algorithm 1 except for updating every two steps in step (ii) using Equation (9), as we optimize $a_{i}\left(t\right)$ and $a_{i}\left(t+1\right)$ in one loop. As shown in Equations (10) and (11), to calculate the probability that node *i* has state 1 at time t+2, we have to list all combinations of nodes leading to having sum of neighbouring states from 0 to $k_{i}$. Therefore, the time complexity for calculating Equation (11) is $O(k_{i}!)$ and will grow exponentially if we look into more than two steps ahead. As it will become infeasible to calculate the combinatorics for more than two steps ahead for large networks, in this paper, we only consider to look one or two steps ahead.

## 4. Results

In this section, our focus is on exploring the best strategies of controller *A* who aims to accelerate the opponent-strategy reconstruction process by optimally allocating its budget to minimize the variance of estimators of controller *B*’s targeting. In order to gain some first intuition about how the budget allocations influence the inference of the opponent’s strategy, we start our analysis by exploring the dependence of variance of MLE on different budget allocations in the equally targeting scenario in Section 4.1. These results also provide a benchmark for later comparison to our optimization heuristics. Next, to investigate the efficiency of the one-step-ahead and two-step-ahead optimization algorithms, we proceed with a numerical exploration of the performance of these two algorithms in Section 4.2 and Section 4.3, respectively. In more detail, we start with using the one-step-ahead and two-step-ahead algorithms to infer opponent’s control at a single node, and then extend the above setting to optimizing multiple nodes with the aim to minimize the sum of variance. To further investigate the dependency of the optimal budget allocations for inference acceleration on network heterogeneity, we carry out detailed numerical experiments based on uncorrelated random scale-free networks with power-law degree distribution $p_{k}\propto k^{-\lambda }$ constructed according to the configuration model [42]. Here, *k* is the node’s degree, and λ indicates the degree exponent. After that, in Section 4.4, we propose an algorithm called optimally equally targeting, which has reduced time complexity compared with the two-step-ahead algorithm at the cost of very little performance loss.

### 4.1. Opponent Strategy Inference in the Equally Targeting Scenario

**Figure 2:**
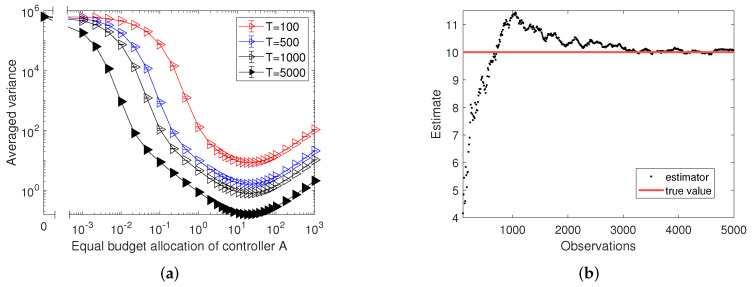
Panel (**a**) shows the dependence of averaged variance of estimators for controller *B*’s budget allocations over all agents on the budget allocations of controller *A*. Differently coloured curves correspond to different lengths of the observation periods *T*. The results are based on 100 repetitions of the experiment on random regular networks with N=1000 nodes and average degree $\langle k\rangle =10$, and we use a setting in which controller *B* targets all nodes equally with budget b=10. Error bars indicate 95% confidence intervals. Panel (**b**) shows an example of one realization of the evolution of the estimator ${\hat{b}}_{1}$ over increasing numbers of observations. The true value of controller *B*’s budget allocation is presented by the red line.

We start with exploring the influence of budget allocations on the variance calculated from Equation (7) in the equally targeting scenario where all nodes are targeted with the same budget allocation. To proceed, in Figure 2a we present numerical results for the dependence of the averaged variance over all agents in random regular networks on the varying budget allocations by the controller *A* for different observation periods *T*. In more detail, in panel (a) of Figure 2, we observe a concave and asymmetric shape of the dependence of the averaged variance on the budget allocated by controller *A*, and clear minimum values of averaged variance can be identified for curves of different observation periods *T*. Moreover, the x-axis of Figure 2a starts from 0, which is identical to the scenario of no interference from controller *A*. In this scenario, agents will align with controller *B* after the first few updates and keep their opinions unchanged thereafter. As information is only gained in flips, estimation under the scenario of no interference is almost impossible. Similarly, extremely small or large allocations (e.g., allocations less then $10^{-1}$ or bigger than $10^{2}$) will cause difficulties in inferring the opponent’s strategy as agents keep their opinions static in most updates. Further to the comparison of curves of different observation periods *T* in Figure 2a, we find that, with the increase of the length of observation periods, the variance of the estimator decreases monotonically. In other words, a more accurate estimator will be obtained after a longer observation period, which is consistent with our analysis in Equations (4) and (5) where the variance will decrease monotonically with the increase of observational data. Additionally, we present the convergence of the maximum likelihood estimation for the opponent budget inference in Figure 2b by showing the dependence of the estimator of MLE on updates. With the increase of the number of observations, the estimator is approaching the true value.

### 4.2. Results for the One-Step-Ahead Optimization

**Figure 3:**
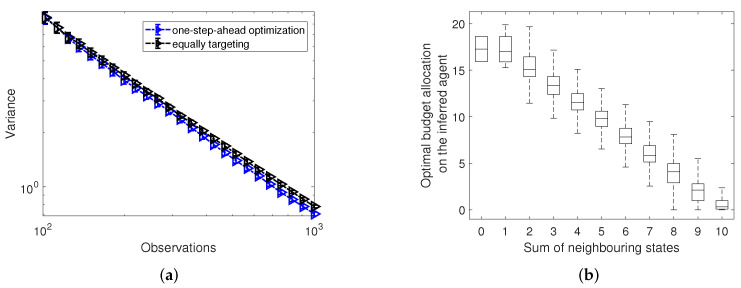
Panel (**a**) compares the variance at a single inferred agent calculated by the one-step-ahead optimization with the variance calculated by the equally targeting strategy with increasing numbers of observations. Error bars in panel (**a**) present 95% confidence intervals. Panel (**b**) shows the dependence of the optimal budget allocations $a_{i}^{*}\left(t\right)$ over updates t=100 to t=1000 calculated by the one-step-ahead optimization in panel (**a**) on the sum of neighbouring states $\sum_{j}w_{ij}s_{j}\left(t\right)$, where *i* indicates the inferred agent *i*. Data in panel (**b**) is organized as box plots, where the central horizontal lines represent the median and the bottom and top box edges are for the 25th and 75th percentiles. The whiskers extend to the maximum or minimum data points. Results in both panels (**a**,**b**) are based on random regular networks with N=1000 nodes and average degree $\langle k\rangle =10$, and are averaged over 100 realizations of the experiment. Controller *B* targets all nodes equally with budget 10 and except for the inferred node, controller *A* targets all the other nodes with budget 20.

To test the efficiency of the one-step-ahead optimization algorithm (see Algorithm 1), we start with exploring the optimal budget allocation for a single agent *i* according to Equation (8) with the aim of minimizing the expected variance step-wisely of the inferred agent. In more detail, in Figure 3a, we compare the variance of MLE calculated by the one-step-ahead optimization from Equation (8) with the variance of the estimator obtained after applying the equally targeting strategy based on random regular networks. We find that, compared to the case of equally targeting, the one-step-ahead optimization algorithm achieves only a slight improvement in speeding up the convergence of the estimate (see the marginal difference in the dependence of variance on the number of observations in Figure 3a). Nevertheless, in order to shed light on the targeting strategy of *A*, in Figure 3b, we further plot the dependence of the optimal budget allocations of controller *A* calculated by the one-step-ahead optimization averaged over updates t=m to t=T on the sum of neighbouring states $\sum_{j}w_{ji}s_{j}\left(t\right)$, where *i* represents the inferred agent. Note that, as depicted in Algorithm 1, *m* represents the number of initialized updates before calculating the variance, and here we assign it as 100. As a result, we observe a clear pattern of the dependence of optimized budget allocations on the sum of neighbouring states: the larger the sum of neighbouring states, the lower the optimized budget allocation. In other words, to speed up estimates, controller *A* tends to target node *i* whenever all the node’s neighbours differ from controller *A*.

**Figure 4:**
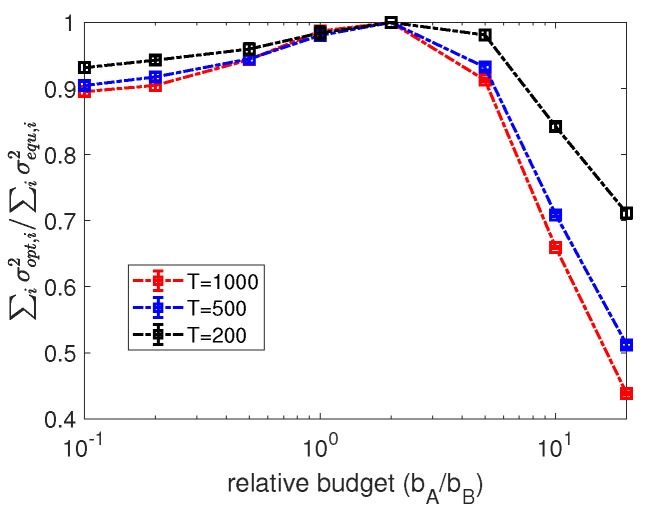
Relative sum of variance $\sum_{i}\sigma_{opt,i}^{2}/\sum_{i}\sigma_{equ,i}^{2}$ achievable by the one-step-ahead optimization compared to the equally targeting strategy for varying relative budgets $b_{A}/b_{B}$. Differently coloured curves correspond to different lengths of the observation periods *T*. Results are based on 100 repetitions of the experiment on scale-free networks with power-law degree distribution of degree exponent λ=1.6, network size N=1000, average degree $\langle k\rangle =6$ and are constructed according to the configuration model. We use a setting in which controller *B* targets all nodes with allocations randomly sampled from a uniform distribution, and the budget allocation by controller *B* per node on average is 10. Error bars indicate 95% confidence intervals.

In the following, we further generalize the setting of attempting to infer the targeting of the *B*-controller at a single node to attempting to infer the targeting of the *B*-controller on all nodes. As a measure for the quality of estimates we use the sum of the variance of estimates at individual nodes and hence we aim at minimizing the sum of the variance of estimators for all agents. By extending Equation (8), we have

(12) $$\begin{array}{c} \begin{array}{cc} \; & \left\{\overset{N\text{ agents in the network}}{\overset{︷}{\;a_{1}^{*}\left(t\right),\ldots ,a_{N}^{*}\left(t\right)}}\;\right\}=\arg\min\sum_{i=1}^{N}{\hat{\sigma }}^{2}\left({\hat{b}}_{i},t+1\right)=\arg\min\sum_{i=1}^{N}-{\left[I\left({\hat{b}}_{i},t\right)+{\hat{\Psi }}_{i}\left(t\right)-{\hat{\beta }}_{i}\left(t\right)\right]}^{-1}\\ \; & \mathrm{subject}\;\mathrm{to}\\ \; & \;\;\;a_{1}^{*}\left(t\right)+\cdots +a_{N}^{*}\left(t\right)\leq b_{A} \end{array} \end{array}$$

where ${\hat{\Psi }}_{i}\left(t\right)={\left(a_{i}\left(t\right)+k_{i}+{\hat{b}}_{i}\right)}^{-2}$, ${\hat{\beta }}_{i}\left(t\right)={\left[\left(k_{i}-\sum_{j}w_{ji}s_{j}\left(t\right)+{\hat{b}}_{i}\right)\left(a_{i}\left(t\right)+k_{i}+{\hat{b}}_{i}\right)\right]}^{-1}$, and $a_{i}^{*}\left(t\right)$ stands for the optimized budget allocation for agent *i* by the controller *A*. Note that the sum of the optimized budget allocations should be subject to the budget constraint, denoted as $a_{1}^{*}\left(t\right)+\cdots +a_{N}^{*}\left(t\right)\leq b_{A}$ in Equation (12). Similar to Figure 3a, in Figure 4, we explore the one-step-ahead optimization algorithm in the scenario of minimizing the sum of variance of estimators in comparison with the equally targeting scenario for varying relative budgets $b_{A}/b_{B}$. In more detail, the improvement achieved by the one-step-ahead optimization is represented by the relative values of the ratios of the sum of variance $\sum_{i}\sigma_{opt,i}^{2}/\sum_{i}\sigma_{equ,i}^{2}$, where $\sum_{i}\sigma_{opt,i}^{2}$ denotes the sum of variance of estimators calculated by the one-step-ahead optimization and $\sum_{i}\sigma_{equ,i}^{2}$ stands for the sum of variance by the equally targeting strategy. After a careful inspection of Figure 4, we find that the one-step-ahead optimization can achieve a considerable improvement in reducing the variance compared with the equally targeting scheme if the active controller has much more budget than its opponent (i.e., $b_{A}\gg b_{B}$). In other settings the one-step-ahead optimization only makes a slight improvement in minimizing the sum of variance, especially when the active controller has almost the same amount of available budget as its opponent. This indicates that the strategic allocation is more critical in accelerating the inference, if the active controller has more resources. In addition, we also find that with an increase in the length of the observation period, the relative values of sum of variance $\sum_{i}\sigma_{opt,i}^{2}/\sum_{i}\sigma_{equ,i}^{2}$ decreases. In other words, more benefits can accrue from the one-step-ahead optimization the longer the period of observation.

**Figure 5:**
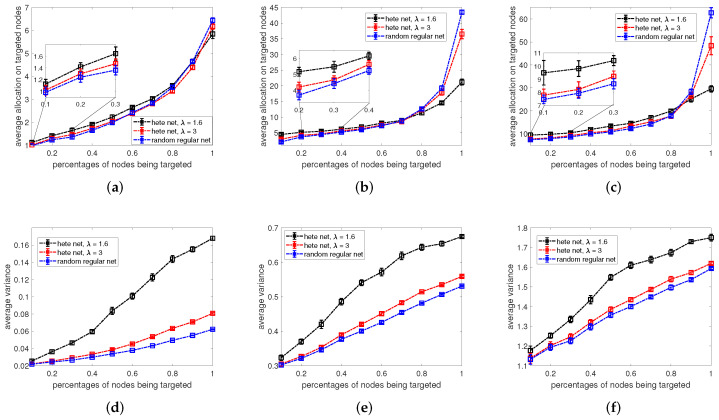
Panels (**a**–**c**) and (**d**–**f**) show the dependence of optimized average allocations for one-step-ahead optimization and corresponding normalized sum of variance of estimates on percentages of nodes being targeted. We use a setting in which controller *B* targets certain percentages of nodes with allocations randomly sampled from a uniform distribution, and the budget allocation per node on average by controller *B* is 1 (**a**,**d**), 5 (**b**,**e**), and 10 (**c**,**f**). The average allocations or variance are calculated by adding up the optimized allocations or variance for a certain percentage of targeted nodes and then divided by the number of nodes targeted. Black and red curves correspond to networks constructed according to the configuration model with power-law degree distribution of exponent λ=1.6 and λ=3, respectively. Blue curves represent random regular networks. Results are based on 20 repetitions of the experiment on networks with size N=1000, average degree $\langle k\rangle =6$. Error bars indicate 95% confidence intervals.

Above, in Figure 4, we consider a scenario in which all agents in the network are subject to the control of the controller *A*, and the active controller wants to infer the budget allocations of its opponent over the entire network. However, in many real-world scenarios such as marketing, the controllers only focus on a subset of agents in the network, e.g., those who are most likely to buy their products. Inspired by this, we further assume that the controller *A* only distributes its budget among the certain fraction of agents targeted by controller *B* and tries to minimize the sum of variance among these agents. Additionally, we are also interested in the implications of network structure on the opponent strategy inference. Therefore, in Figure 5, we show the dependence of average variance achievable by the one-step-ahead algorithm on the percentage of nodes being targeted by controller *B*. The results in Figure 5 are compared among regular random networks and scale-free networks with power-law degree exponents λ=1.6 and λ=3. Here, the average variance is calculated only within the targeted nodes, i.e., the average variance equals to the sum of variance of the inferred agents divided by the number of nodes being targeted. More specifically, results for the dependence of averaged optimized budget allocation by the one-step-ahead optimization on varying percentages of nodes being targeted are given in Figure 5a–c, where panels correspond to controller *B* targeting nodes with allocations randomly sampled from a uniform distribution, and the budget allocation per node on average is 1, (Panel (a)), 5 (Panel (b)) and 10 (Panel (c)). For the corresponding settings the dependencies for the optimized average variance are presented in Figure 5d–f. From Figure 5a–f, we obtain the following observations about the the strategic allocations of the active controller. First, we see similar patterns in Figure 5a–c where with an increase in the number of agents being targeted, on average more resources are needed for the controller *A* to perform optimal inference. Depending on the budget availability of the controller *B*, the optimized controller *A* allocates more resources on each targeting node on average accordingly as $b_{B}$ increases for the same amount of nodes being targeted, e.g., comparing the y-axis of the blue line in panel (a) to the blue line in panel (c). Meanwhile, in Figure 5d–f, as budget allocations from the opponent increase, the variance of the estimators rises. This is consistent with the analytical results in Equation (6), which indicates that a higher value of budget allocation is harder to be predicted. Second, by comparing the curves of optimized budget allocations for different types of networks, we find that, only when a large portion of nodes are targeted then there is significant difference in the optimized budget allocations among networks with different degree distributions. Otherwise, the optimized budget allocations are fairly close for networks with different degree heterogeneity. However, if we zoom in and compare the ordering of curves in Figure 5a–c for small number of nodes being targeted with large number of nodes being targeted, we find that there are two regimes for the strategy of the optimized controller depending on the network heterogeneity. In more detail, the optimized controller will allocate more resources on a more heterogeneous network than on a less heterogeneous network if only a small portion of nodes are targeted. The opposite holds if a large number of nodes are under control of the active controller. Third, in Figure 5d–f, we find that more degree-heterogeneous networks always have higher average variance, i.e., opponent strategies are the more difficult to infer the more heterogeneous the network.

In Figure 5, we always assume that the opponent targets nodes with allocations randomly sampled from a uniform distribution. However, we are also interested in the effects of the opponent’s strategy on the predictability of the optimized controller. Therefore, in the following, we further explore the strategic allocations of the active controller based on different budget allocation strategies of its opponent. To proceed, we consider a scenario in which the opponent allocates resources as a function of the node’s degree. More specifically, suppose the opponent generates random numbers $r_{i}$(1≤i≤N) from the interval of $\left[0,k_{i}^{\alpha }\right]$ for each of the *N* nodes, where $k_{i}$ is the degree of node *i* and the exponent α indicates the varying strategies of the opponent. For instance, for α=0 an opponent would allocate independent of degree based on uniform random numbers, for α=1 the opponent would on average allocate proportional to degree, whereas for α=−1 average allocations would be inversely proportional to degree. By then normalizing the random numbers $r_{i}$ to satisfy the budget constraint of controller *B*, we obtain different budget allocations $b_{i}$.

**Figure 6:**
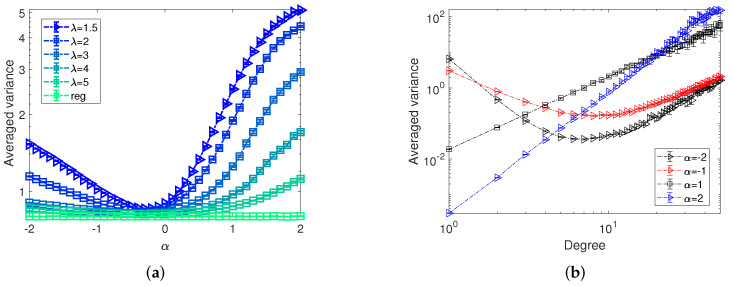
Panel (**a**) shows the dependence of averaged variance obtained by one-step-ahead optimization on the opponent strategy exponent α, where the budget allocation of the controller *B* is generated proportional to the random number within the interval $\left[0,k_{i}^{\alpha }\right]$. Different colours correspond to different degree exponents λ of the scale-free networks and reg corresponds to random regular graphs as indicated in the legend. Results are based on 100 repetitions of the experiment on networks with size N=1000, average degree $\langle k\rangle =10$. Error bars indicate 95% confidence intervals. For the setting corresponding to λ=1.5 in panel (**a**), panel (**b**) shows the dependence of corresponding averaged variance of networks on the node’s degrees for varying opponent strategy exponents α.

In Figure 6, we plot the dependence of optimized averaged variance obtained by the one-step-ahead optimization on the opponent strategies represented by the varying exponents α. We observe a concave shape of the averaged variance along with the changing exponents α, with minima near α=0. For all settings of α we generally also observe larger average variance the more heterogeneous the networks. To proceed, Figure 6b shows the dependence of averaged variance on nodes’ degree. We find that, generally, nodes with larger degree are more difficult to predict as the averaged variance of estimators are larger. In a similar vein, nodes being allocated larger budgets by the opposing controller are also harder to predict which can be seen from the curves for α=−1,−2, as in this setting low degree nodes have larger averaged variance than the high degree nodes.

### 4.3. Results for the Two-Step-Ahead Optimization

**Figure 7:**
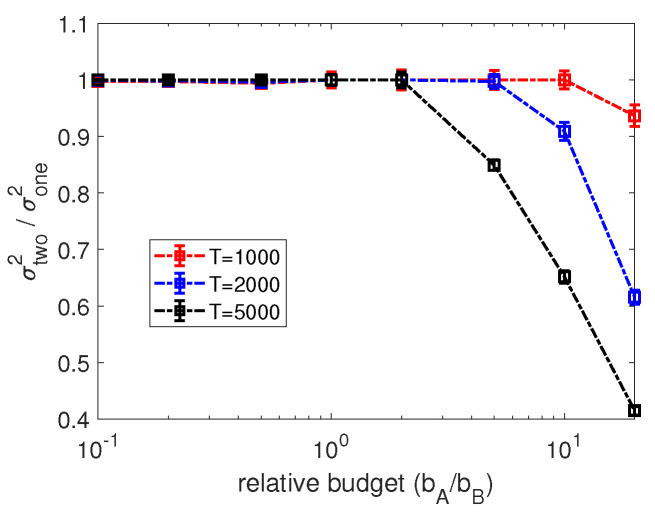
Relative variance $\sigma_{two}^{2}/\sigma_{one}^{2}$ of the estimate for a single inferred node achievable by the two-step-ahead optimization compared to the one-step-ahead scheme for varying relative budgets $b_{A}/b_{B}$. Differently coloured curves correspond to different lengths of the observation periods *T*. Results are based on 100 repetitions of the experiment on random regular networks with size N=1000, average degree $\langle k\rangle =10$. Controller *B* targets all nodes equally with allocation 10 and except the inferred node, controller A targets all the other nodes with budget 10. Error bars indicate 95% confidence intervals.

In this section, we proceed with testing the efficiency of the two-step-ahead optimization algorithm. Similar to Section 4.2, we start by minimizing the variance of a single agent using the two-step-ahead optimization over random regular networks with network size N=1000 and average degree $\langle k\rangle =10$. In more detail, in Figure 7, we compare the variance of the estimator on a single node *i* calculated by two-step-ahead heuristics with the one-step-ahead optimization for varying relative budget constraints $b_{A}/b_{B}$ based on different observation periods *T*. Note that, for the two-step-ahead optimization, we set the constraint $b_{A}$ separately for time steps *t* and t+1. Therefore, assigning the optimized allocation obtained by the two-step-ahead algorithm at time steps *t* and t+1 as $a_{i}^{*}\left(t\right)$ and $a_{i}^{*}\left(t+1\right)$, we have $a_{i}^{*}\left(t\right)\leq b_{A}$ and $a_{i}^{*}\left(t+1\right)\leq b_{A}$. By observing the results in Figure 7, we find that, similar to the results of Figure 4, the two-step-ahead optimization can achieve a considerable improvement in reducing the variance compared with the one-step-ahead scheme only if the active controller has much more budget than its opponent.

**Figure 8:**
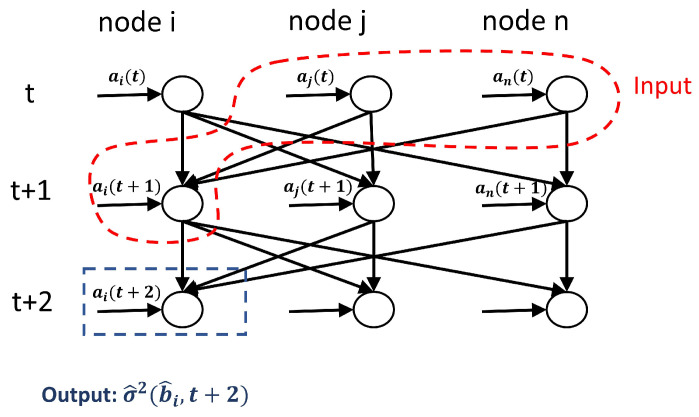
Schematic illustration of a variant of the two-step-ahead optimization in the context of optimizing the budget allocations for an inferred node *i* and its neighbourhood. Here, we assume that node *j* and node *n* are the two neighbours of node *i*. Each column presents the state dynamics of a node from time *t* to t+2. The arrows indicate interactions between nodes and controller *A* which determine the transition probabilities. For example, the state of node *i* at time t+1 depends on the states of node *j* and node *n* at time *t*, as well as the budget allocation $a_{i}\left(t\right)$. Therefore, there are arrows from node *j* and node *n* at time *t* point to node *i* at time t+1, as well as a horizontal arrow indicating the budget allocation from controller *A* at time *t* labelled by $a_{i}\left(t\right)$. The state of node *i* at time t+2 is determined by $a_{i}\left(t+1\right)$ and states of node *j* and *n* at time t+1. To influence states of node *j* and *n* at time t+1, we have to change the budget allocations at time *t*. Therefore, the inputs of the optimization of minimizing the variance of node *i* by optimizing the budget allocations for the inferred node and its neighbourhood are $a_{i}\left(t+1\right)$, $a_{j}\left(t\right)$ and $a_{n}\left(t\right)$ (see the variables circled by the red dashed line).

Notice that, in the log-likelihood function of Equation (3) composed of transition probabilities $Pr\left(s_{i}\left(t+1\right)=1\right)=\frac{a_{i}\left(t\right)+\sum_{j}w_{ji}s_{j}\left(t\right)}{a_{i}\left(t\right)+b_{i}+k_{i}}$ and $Pr\left(s_{i}\left(t+1\right)=0\right)=\frac{b_{i}+\sum_{j}w_{ji}\left(1-s_{j}\left(t\right)\right)}{a_{i}\left(t\right)+b_{i}+k_{i}}$, the budget allocation from the controller *A* (i.e., $a_{i}\left(t\right)$) is not the only determinant that influences the inference of $b_{i}$. Instead, the sum of the neighbouring states $\sum_{j}w_{ji}s_{j}\left(t\right)$ of the inferred node *i* is also taken into consideration when inferring $b_{i}$. Therefore, a natural extension for the above scenario of minimizing the variance of a single node by only targeting that inferred node is to optimize the inference at the focus node by targeting it and its neighbours. For clarification, a schematic illustration of optimizing the budget allocations for the inferred node and its neighbourhood to minimize the variance of the central node is given in Figure 8. In more detail, in Figure 8, we have shown that to minimize the variance of the estimator ${\hat{b}}_{i}$ for node *i* at time step t+2 (marked as output), we have to optimize the budget allocation for the inferred node one step ahead and its neighbours two steps ahead (circled in red). A reason for optimizing the budget allocation of the neighbouring nodes two step ahead is that by doing so, we could influence the sum of neighbouring states at time t+1, and afterwards the variance of the inferred node at time t+2. Therefore, the optimization in this scenario can be viewed as a variant of the two-step-ahead optimization of Equation (9), and the objective function is given by

(13) $$\begin{array}{c} \begin{array}{cc} \; & \left\{a_{i}^{*}\left(t+1\right),\overset{\text{neighbours of node }i}{\overset{︷}{a_{j}^{*}\left(t\right),\ldots ,a_{n}^{*}\left(t\right)}}\right\}=\arg\min{\hat{\sigma }}^{2}\left({\hat{b}}_{i},t+2\right)\\ & =\arg\min-{\left[Pr\left(s_{i}\left(t+2\right)=1\right)\times {\hat{\Psi }}_{i}\left(t+1\right)+Pr\left(s_{i}\left(t+2\right)=0\right)\times {\hat{\Upsilon }}_{i}\left(t+1\right))+I\left({\hat{b}}_{i},t+1\right)\right]}^{-1}\\ \; & \mathrm{subject}\;\mathrm{to}\\ \; & \;\;\;a_{i}^{*}\left(t+1\right)+a_{j}^{*}\left(t\right)+\cdots +a_{n}^{*}\left(t\right)\leq b_{A} \end{array} \end{array}$$

where $Pr(s_{i}\left(t+2\right)=1)$ and $Pr(s_{i}\left(t+2\right)=0)$ represent the probability for node *i* to have opinion 1 and 0 at step t+2, respectively. Moreover, ${\hat{\Psi }}_{i}\left(t+1\right)$ and ${\hat{\Upsilon }}_{i}\left(t+1\right)$ are consistent with the definition in Equation (9). Inserting Equations (10) and (11) into Equation (13) yields the full expression. Here, we use the interior-point method for the optimization of Equation (13), and the corresponding time complexity to obtain $a_{i}^{*}\left(t+1\right),a_{j}^{*}\left(t\right),\cdots ,a_{n}^{*}\left(t\right)$ is $O(k_{i}!T)$, where $k_{i}$ is the degree of node *i* and *T* is the length of the observation period.

**Figure 9:**
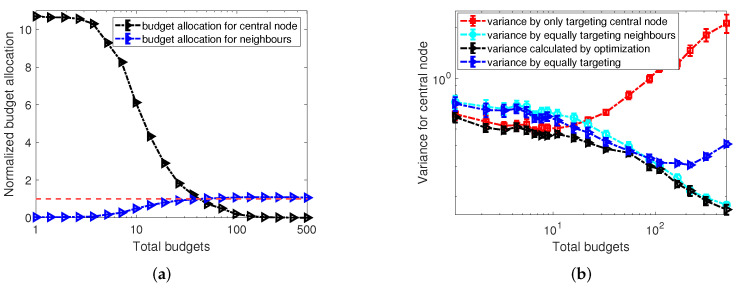
Panel (**a**) shows the dependence of normalized budget allocations ${\tilde{a}}_{j}=\frac{a_{j}\left(k_{i}+1\right)}{b_{A}}$ after the first T=1000 updates calculated by Equation (13). The black triangles are the budget allocations for each neighbouring node where differences in allocations to different neighbours are characterized by error bars. Panel (**b**) shows the dependence of variance of MLE of the central node on varying total budgets at update T=1000 based on four budget allocation strategies: only targeting the central node (red squares), equally targeting neighbours only (red circles), optimization described in Equation (13) (black triangles), and equally targeting (blue triangles). The results are based on 20 realizations of random regular networks with 1000 nodes and average degree $\langle k\rangle =10$. controller *B* targets all nodes equally with budget 5, and except for the inferred node and its neighbours, controller *A* targets all the other nodes with budget 5. Error bars indicate 95% confidence intervals.

To distinguish differences in allocations made by the optimized controller to the central inferred node and on its neighbours, we partition the budget allocations for these two types of nodes in two groups and normalize by the average budget allocation to any node. We thus have ${\tilde{a}}_{i}=\frac{a_{i}\left(k_{i}+1\right)}{b_{A}}$ for the central node and for the neighbouring nodes j∈Nei(i) we have ${\tilde{a}}_{j}=\frac{a_{j}\left(k_{i}+1\right)}{b_{A}}$. In Figure 9a, we show the dependence of the normalized optimized budget allocations to the central node and its neighbours on varying budget availability $b_{A}$ of controller *A*. We clearly observe two regimes of budget allocations for the central node and its neighbours. For small enough budget availability to A, all of the resources will be focused on only the central node. However, with an increase of the budgets available to the optimized controller, more and more resources will be diverted to its neighbours until a crossing point is reached. Finally, for large enough budget $b_{A}$, only the neighbouring nodes will be targeted.

Motivated by the optimized schemes of budget allocations for the central and neighbouring nodes in the context of extremely small and large budget constraints in Figure 9, we propose two other heuristics. One is allocating all of the resources on the central node and leaving its neighbours un-targeted. The other is targeting the neighbouring nodes equally, but leaving the central node empty. In Figure 9b, we compare the variance for the central node calculated by only targeting the central node (represented by red squares), by only equally targeting the neighbours (light blue circles), and by the optimization of Equation (13) (marked as black triangles), and also the strategy of equally targeting all node (including the central node and its neighbours) as the benchmark. The results in Figure 9b are consistent with what we observe in Figure 9a. Although the optimized strategy has the best performance in reducing the variance of the central node for all values of budget constraints compared with the three other strategies in Figure 9b, for small total budgets, the variance calculated by strategy of only targeting the central node is close to the variance by the optimized strategy. Meanwhile, for large total budgets, the strategy of only equally targeting the neighbours has almost the same performance as the optimized strategy. Our finding suggests that, instead of applying the optimization of Equation (13) whose time complexity is $O(k_{i}!T)$, we could substitute it with simple heuristics of targeting the central node or neighbours only without sacrificing much in performance.

In the following, we extend the scenario of minimizing the variance of a single node to minimizing the sum of variance of estimators over the whole network with the two-step-ahead heuristics. In this context, we have

(14) $$\left[\begin{array}{ccc} a_{1}^{*}\left(t\right) & \cdots & a_{N}^{*}\left(t\right)\\ a_{1}^{*}\left(t+1\right) & \cdots & a_{N}^{*}\left(t+1\right) \end{array}\right]=\arg\min\sum_{i=1}^{N}{\hat{\sigma }}^{2}\left({\hat{b}}_{i},t+2\right)=\arg\min\sum_{i=1}^{N}-{I({\hat{b}}_{i},t+2)}^{-1}$$

**Figure 10:**
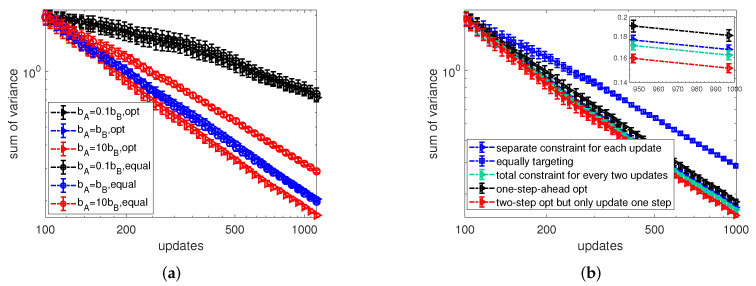
Panel (**a**) compares the sum of expected variance of MLE calculated by two-step-ahead optimization with total constraint for every two updates (marked by “opt”) and equally targeting strategy (marked by “equal”) based on three different relative budget constraints $b_{A}/b_{B}=0.1$, $b_{A}/b_{B}=1$ and $b_{A}/b_{B}=10$ with increasing numbers of observations. Panel (**b**) compares the sum of expected variance of MLE calculated by 5 different methods under the control setting of $b_{A}=10b_{B}$. Here, ’two-step opt but only update one step’ stands for using two-step-ahead algorithm but only update the budget allocation for the next step. ’one-step-ahead opt’ presents the one-step-ahead algorithm. Results are based on 20 repetitions of the experiment on ring networks with size N=10. Controller *B* targets nodes with allocations randomly sampled from a uniform distribution, and the budget allocation per node on average by controller B is 2. Error bars indicate 95% confidence intervals.

Generally, there are two options to set the constraint for the two-step-ahead optimization in the context of minimizing the sum of variance for the entire network. One is to set the budget constraint separately for each update, i.e., $a_{1}\left(t\right)+\cdots +a_{N}\left(t\right)\leq b_{A}$, referred to as *SCEU* in the following. The other is to set a total constraint for every two updates where $a_{1}\left(t\right)+\cdots +a_{N}\left(t\right)+a_{1}\left(t+1\right)+\cdots +a_{N}\left(t+1\right)\leq 2b_{A}$, referred to as *TCEW*. Due to the exponential time complexity of the two-step-ahead algorithm with respect to the network size *N*, we start our analysis of the optimal budget allocations for minimizing the sum of variance by numerical experiments conducted on a small ring graph of N=10 nodes. In more detail, Figure 10a compares the sum of variance calculated by the two-step-ahead method (marked as “opt”) and the equally targeting strategy (marked as “equal”) for varying budget constraints $b_{A}/b_{B}=0.1,1,10$ with increasing numbers of observations. Moreover, we use a setting in which controller *B* targets all nodes with allocations randomly sampled from a uniform distribution, and the budget allocation per node on average is 2. After a careful inspection of Figure 10a, we obtain a similar observation as in the case of optimizing one node by the two-step-ahead optimization in Figure 7. The two-step-ahead optimization can make significant improvement in reducing the sum of variance of estimators compared with the equally targeting strategy only when the active controller has much more budget than its opponent. This suggests the two-step-ahead optimization is more effective if the optimized controller have more available resources.

Since the substantial improvement in minimizing the sum of variance by the two-step-ahead optimization has only been observed when controller *A* is in budget superiority, we only investigate this scenario further. In the following, we proceed by comparing the sum of variance obtained by applying the one-step-ahead optimization, the two-step-ahead optimization and the equally targeting strategy in Figure 10b under the same network setting as Figure 10a and control setting of $b_{A}/b_{B}=10$. From Figure 10b, we find that the “two-step opt but only update one step” heuristics which uses a two-step-ahead algorithm but only updates the budget allocation for the next step has the best performance in the scenario of controller *A* having more budget than controller *B*. An explanation for this is: by optimizing two steps ahead, this heuristics accounts for the indirect influence between nodes, while, by only updating one step ahead, the controller can adjust its prediction of two-step-ahead variance after one update, as well as adjust its budget allocation for the next step. Additionally, even though SCEU and TCEW are separated by different ways of imposing the budget constraint, there is no significant difference in the sum of variance obtained by these two methods. Moreover, as expected we also observe that the one-step-ahead method has the worst performance among all the heuristic methods, but is nevertheless still better than the equally targeting strategy.

### 4.4. Optimally Equally Targeting

As seen in Figure 4 and Figure 10, the one-step-ahead and two-step-ahead optimization algorithms have better performance in reducing the variance of estimators of the opponent’s budget allocations compared with the equally targeting strategy. However, the cubic and exponential time complexity of the one-step-ahead and two-step-ahead optimization algorithms in terms of network size *N* make them unsuitable for application to large-size networks. To address this issue of scalability of the one-step-ahead and two-step-ahead optimizations, we propose a new heuristic algorithm named optimally-equally-targeting strategy (*OETS*), where we attempt to find an optimal equal allocation for all nodes in the network. Specifically, the heuristics of the optimally-equally-targeting strategy is motivated by the observations in Figure 9b which shows that putting too many resources on the inferred agent only will deteriorate the accuracy of the inference. Moreover, Figure 4 and Figure 10 also indicate that only limited improvement of variance reduction will be achieved by the one-step-ahead and two-step-ahead algorithms compared with the equally targeting strategy when the active controller has less or equal budgets compared to its opponent.

Formally, the objective function of the OETS is given by

(15) $$a^{*}=\arg\min\sum_{i=1}^{N}{\hat{\sigma }}^{2}\left({\hat{b}}_{i},T\right)=\arg\min-\sum_{i=1}^{N}{\left[\sum_{t=0}^{T-1}({\hat{\Psi }}_{i}\left(t\right)-{\hat{\beta }}_{i}\left(t\right))\right]}^{-1}\;\left(0\leq a^{*}\leq b_{A}/N\right)$$

where $a^{*}$ is the optimal budget allocation for all nodes to achieve a minimum sum of variance after *T* observations, $b_{A}$ is the budget constraint for controller *A*, ${\hat{\Psi }}_{i}\left(t\right)={\left(a+k_{i}+{\hat{b}}_{i}\right)}^{-2}$, ${\hat{\beta }}_{i}\left(t\right)={\left[\left(k_{i}-\sum_{j}w_{ji}s_{j}\left(t\right)+{\hat{b}}_{i}\right)\left(a+k_{i}+{\hat{b}}_{i}\right)\right]}^{-1}$. Here, by proposing the optimally-equally-targeting strategy, we have reduced the parameter space from *N* (the one-step-ahead optimization) or 2N (the two-step-ahead optimization) to 1 and the time complexity to O(T) without sacrificing much of the performance.

**Figure 11:**
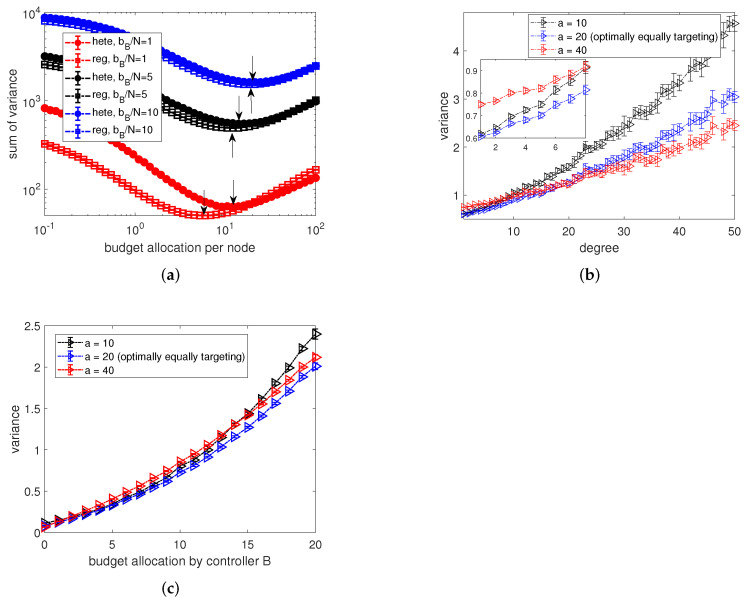
Panel (**a**) shows the dependence of sum of variance of estimators for controller *B*’s budget allocations over all agents on the equal budget allocations of controller *A* at update T=1000. Differently coloured curves correspond to varying budget constraints of controller *B*, e.g., the red lines marked with $b_{B}/N=1$ indicates controller *B* targets each node with 1 on average. Circles and squares correspond to scale-free networks with degree exponent λ=1.6 and random regular networks, respectively. The results are based on 20 repetitions of the experiment on networks with N=1000 nodes and average degree $\langle k\rangle =6$. Controller *B* targets nodes with allocations randomly sampled from a uniform distribution. Controller *A* targets all nodes equally. Panels (**b**,**c**) present the dependence of the corresponding variance of estimators achieved by equally targeting each node with allocation 10, 20, and 40 in Panel (**a**) on nodes’ degree (**b**) and budget allocations (**c**) by the opponent for the scale-free networks with degree exponent λ=1.6 under the context of $b_{B}/N=10$. Note that a=20 is the optimal budget allocation for equally targeting obtained from Panel (**a**) (the minimum point) for $b_{B}/N=10$. In Panel (**c**), we group the value of x axis into bins with width 1 and lower limits are inclusive, e.g., [0, 1). Error bars indicate 95% confidence intervals.

To explore how the budget availability and network structures affect the optimally equally targeting strategy, in Figure 11a, we show the corresponding sum of variance of MLE for varying equal budget allocations by the active controller on networks with different heterogeneity in the context of the opponent targeting each node with average budget 1, 5 and 10. Additionally, controller *B* targets nodes with allocations randomly sampled from a uniform distribution. Note that the optimally equally targeting strategy for each scenario in Figure 11a is marked by the arrows. By comparing curves for networks with different degree heterogeneity in Figure 11a, we find that, similar to the results of Figure 5, the variance of estimators for random regular networks is always smaller than that for the heterogeneous networks.

As the main difference of networks of different types is the degree distribution, to explore how the degree of nodes play a role in OETS, we present the dependence of variance of estimators on nodes’ degrees in Figure 11b. Clearly, we observe a positive relationship between the variance and nodes’ degree. This result has further explained the degree-based heuristics for the link weight prediction in [25] about why the solution obtained from a lower-degree node are preferred. Moreover, with a careful inspection of Figure 11b, we observe two regimes. For low-degree nodes, a large equal allocation by controller *A* (e.g., a=40) will result in a worse performance in predicting the budget allocations. However, for the hub nodes, a larger allocation is preferable in improving the accuracy of the prediction. Furthermore, by comparing the patterns of the dependence for equal budget allocations by controller *A* for a=10,20,40 in Figure 11b, we find that the OETS results from a trade-off. On the one hand, heterogeneous networks have more low-degree nodes, therefore relatively high budget allocations from the controller *A* should be avoided. On the other hand, as the hub nodes normally have much higher variance than low-degree nodes, low budget allocations from the controller *A* is inefficient in minimizing the sum of variance.

Another important factor in the strategy inference is the budget allocation by the opponent. Therefore, in figure (c), we present the dependence of variance on opponent’s budget allocations. Note that, as the budget allocations by the opponent are randomly sampled from a uniform distribution, for ease of observation, we group values into bins with width 1, i.e., $\left\{[0,1),[1,2),\cdots \right\}$ in Figure 11c. Similar to Figure 11b, with on increase of opponent’s budget allocations, the variance of estimates rises monotonically. However, curves for different budget allocations *a* are fairly close and a larger *a* will not result in a lower variance for nodes which are allocated more resources by the opponent.

## 5. Discussion

In this paper, we have proposed an approach to apply network control in the context of a network inference problem. In our setting, an active controller interacts with a process of opinion dynamics on a network and aims to influence the resulting opinion dynamics in such a way that estimates of an opposing controller’s strategy can be accelerated. Existing approaches related to such types of inference problems are often based on the assumption that the inference is performed using given data. In contrast, our approach aims to strategically interfere with the networked dynamics to generate more informative datasets.

By using the variance deduced from the Fisher information as a criterion of inference uncertainty, we have proposed several optimization heuristics. In a first step, in a benchmark scenario in which an active controller can target nodes uniformly by an adjustable amount of influence, we have demonstrated that interference with the system’s dynamics can substantially accelerate the convergence of estimates about opponents. We have then proceeded to develop more sophisticated optimization heuristics, based on step-wise updating of the interference with the dynamics and have shown that such approaches are typically effective if the active controller has a relatively large budget.

Next we have explored the one-step-ahead and two-step-ahead heuristics systematically in a variety of scenarios. First, in a scenario in which the active controller only aims at inference of a single node, we find that only very limited acceleration can be achieved by targeting only this node. However, far more substantial results can be achieved by also targeting the node’s neighbours. For the latter setting we have demonstrated the effectiveness of a simple heuristics, which relies on targeting only the focal node when the controller’s budget is small and only conditionally influencing the focal node’s neighbours when budget availability is large. Conditional targeting of neighbours should be carried out whenever a majority of them are not aligned with the active controller.

Furthermore, we have explored the effectiveness of inference acceleration for networks with varying amounts of degree heterogeneity for different settings of the opponent’s influence allocations. As one might expect, we find that both, predicting opponent influence at nodes with large degrees, and precisely predicting large opponent influence nodes, are difficult. The first is essentially due to the presence if a large changing environment of the node which makes it difficult to distinguish the influence of control from the influence of neighbours. This finding is consistent with results presented in [25] in the context of link inference from static data. The second is due to the effect that large opponent control tends to fix a node in a static state, which makes it difficult to precisely predict the amount of opponent’s influence.

As a consequence of the above, if an opponent targets uniformly at random the inferrability of its influence is strongly related to the number of high-degree nodes on a network. Correspondingly, using our optimization schemes, we find that inference is the more difficult the larger the degree-heterogeneity of a network. The above finding also holds when opponent’s influence with a strength is drawn randomly with inverse proportionality to node degrees. In this case networks with higher degree heterogeneity will also have larger average variance, since they have more low-degree nodes with large opponent influence, which also impedes inference.

Even though the framework we suggest is more general, results of our paper are restricted to analysing accelerating the opponent strategy inference using voting dynamics. However, we believe that the heuristics we propose can also be used in other complex systems with binary-state dynamics such as the Ising model and the susceptible–infected–susceptible model, which we leave for future work. Another limitation of our study is that we only consider opponents with a fixed strategy. Therefore, an interesting line of future enquiry might be to explore the inference acceleration in scenarios in which opponent influence changes dynamically.

## Data Availability

Data will be provided on request from the corresponding author.

## Abbreviations

The following abbreviations are used in this manuscript:

SIS	Susceptible–infected–susceptible
MLE	Maximum-likelihood estimation
SCEU	Setting the budget constraint separately for each update
TCEW	Total constraint for every two updates
OETS	Optimally equally targeting strategy
